# Strengthening Rubber Dentures and Repairing Broken Plates

**Published:** 1878-08

**Authors:** F. E. Howard

**Affiliations:** Genesee, N. Y.


### ARTICLE II.

### *Strengthening Rubber Dentures and Repairing Broken Plates.*

BY F. E. HOWARD, D. D. S., GENESEE, N. Y.

Read before the Seventh District Dental Society of State of New York.

Doubtless we have all experienced difficulty in making repairs in rubber-work, satisfactory to ourselves and patrons, where rubber alone is used. In those cases where plates are slightly cracked, and those broken entirely in two, when repaired with rubber alone, are weak, or clumsy when strong, from the great amount of material used.

Now I propose to show how almost any kind of a fracture may be overcome, with a certainty, by the exercise of a little ingenuity on the part of the dentist, and without thickening the plate, leaving the denture still stronger at the point or place fractured than it was originally.

Rubber, as a general thing, gives good satisfaction to our patrons as a cheap base; but we often feel that additional strength would be desirable, and is often required, in many places where it would be objectionable to have the amount of material necessary for the strength required.

By the method I propose to describe, all rubber dentures can be strengthened and made nearly as strong as an entire metal plate, with comparatively little outlay, but often requiring no small amount of painstaking and nicety of manipulation. But in all communities there are a sufficient number of appreciative people who are willing to pay one, in any department, for honest endeavor to increase their comfort and promote their welfare: and for this class some of you may have an opportunity of applying this method. It is my conviction that it will pay both dentist and patient to give it a trial, in some of the many cases that will be presented, and where it can be applied.

In strengthening new dentures or broken plates, I use gold or platina, either in the form of strips of rolled gold, or wire. This should be roughened or flattened, to suit the circumstances of the case, so that the rubber will take hold of it firmly. In strengthening new cases thinner bands can be used with greater safety than when repairs are to be made upon old work. The whole labial and buccal border may be strengthened, and such places in the palatal portion as are likely to have great strain upon them. In temporary cases, that have been worn a long time, where the alveola has become absorbed to quite an extent, leaving the strain upon the center of the plate, (as the hard palate is unyielding, and brought in direct contact with the plate at this line, under all circumstances,) we often find them broken entirely in two, through the center. Or, perhaps, a patient will insist on leaving one or two teeth because they are sound, (usually cuspid,) and have them fitted around; at their cut-out there is inevitably a weak point in the plate, which, if not strengthened by a strong band, is quite likely to crack, even though left clumsily thick in that quarter. In partial unders, where the molars and bicuspids are to be replaced with artificial ones, a band should connect them, and rest against the lingual surface of the incisors and cuspids. This should be nicely adjusted, and can be fitted to a plaster cast, packed about with rubber, which will make such a denture strong, yet delicate.

In strengthening the alveolar border of uppers, the wire or thin strip must be bent to conform to the general shape of the cast, and packed in with the rubber, calculating to have but very little rubber between the bands and the cast.

By so doing, the case can be dressed down quite thin, and yet be very strong.

I shall only need to illustrate how I would repair a full case broken entirely in two, to make it plain to you all how it is done by this method, or exhibit the case I have with me, to make it more simple. Bring the broken pieces together, as they properly belong, and wax them in their position. Fill the palatal portion with plaster, and allow it to become firm and hard. Remove the wax, now that the pieces are attacked firmly, and, with the engine and a wheel burr, run along the whole length of the break or crack; then sandpaper the polished surface slightly, so that the trace of a lead pencil can be seen; then mark out such places, extending across the broken part, on the palatial portion and labial border, as you desire to have banded and supported with new rubber. Now with the engine and a burr-wheel go over the ground marked out with a pencil; this gives an outline of the section to be cut out. Then, with a coarse round burr, these places are cut almost through, leaving but a thin shell of the old material. Undercuts and dovetails are to be obtained at such places in these cutouts as are strongest, in order to secure the new material.

In small partial cases this work can only be done after the parts are secured in plaster, and put up in the flask.

Now that the cavities for the reception of the new rubber and bands are all prepared, they should be filled with wax a little flush, so as to allow for finishing down nicely.

A piece of gold should be selected for the cutout on the labial border, about one-fourth of an inch longer than is required to fill the cavity in length, and about one eighth of an inch at each end should be bent at a right angle, giving it the form of a bracket. This should be pressed into the wax, and occupy the same position you desire it to have in the rubber; a conical roll of wax should join this, and extend directly down to the cutting-edge of the teeth. One end of this will be at the surface of the plaster in one section, and form the avenue through which the rubber is forced to the place we wish to mend. The projecting ends of the gold band are held by the plaster.

Oil over the whole of this section; adjust the flask rim; fill with plaster; separate the section; remove what wax you can conveniently with the wax-knife, and put the case in water, and have it boil. Now, with a syringe, (I use a common rubber syringe, kept for this purpose alone,) the wax is forced out of every undercut and depression, leaving it perfectly clean. The case is then removed from the water, and such pieces of rolled gold-plate or platina are cut as will drop into the cavities easily on the palatal surface. These are spurred or roughened, that the rubber may take a firm hold. Then, by warming a piece of rubber, make it thin; stretch it across the cavity, and press the band well into the cutout, then cover with another piece. When the places are all filled up, the case is ready to vulcanize.

When the case is removed from the vulcanizer, it is finished off in the usual manner. Occasionally the gold will be seen at different points when finished, but it only serves to show how securely the case has been repaired. It secures to the denture strength that cannot be had in any other way.

Partial plates can be strengthened by this method, and an isolated tooth can be supported and made as strong as though it were one of a section of teeth, by simply cutting a strip of gold and bending it with plyers to conform to the general shape of the cast; imbed it in rubber at its proper place, and vulcanize it in that position.

I have had occasion to strengthen and repair celluloid plates in about the same manner; and by coating the surface desired to be united, with celluloid dissolved or cut in spirits of camphor, they unite into an homogenous body.

By this method of strengthening and repairing celluloid plates, we are enabled to make a success of many cases that would otherwise be weak and doubtful.—*Dental Office and Laboratory.*

